# Correction: Anders et al. First Principle Surface Analysis of YF_3_ and Isostructural HoF_3_. *Materials* 2022, *15*, 6048

**DOI:** 10.3390/ma16144997

**Published:** 2023-07-14

**Authors:** Jennifer Anders, Niklas Limberg, Beate Paulus

**Affiliations:** Institute for Chemistry and Biochemistry, Freie Universität Berlin, Arnimallee 22, 14195 Berlin, Germany

In the original publication [1], there was a mistake in ***Table 2*** and ***Figure 2*** as published. Within Table 2, the coordination numbers of the two stoichiometric terminations of surface (011) have been flipped [showing (011)-1 and (011)-2 with 6,6,8,8 and 7,7,9,9, respectively]. Figure 2 showed the surface of (011)-2 in the first row, third image with the same incorrect coordination numbers of 7,7,9,9 instead of 6,6,8,8. The authors state that the scientific conclusions are unaffected. This correction was approved by the Academic Editor. The original publication has also been updated.

## Figures and Tables

**Figure 2:**
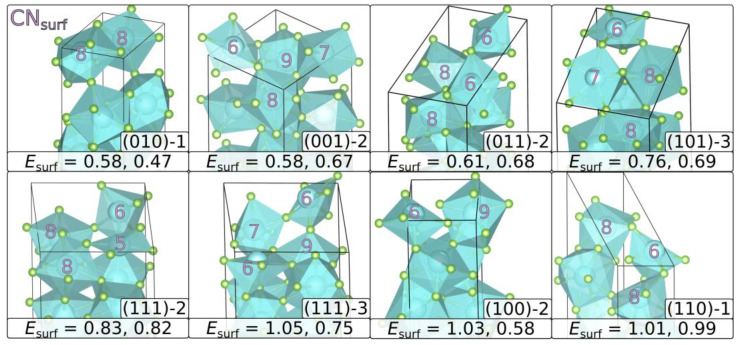
Most stable terminations of the relaxed surface structures: the coordination number of the surface metals (CN_surf_) and the surface energies in J m^−2^ (*E*_surf_) are given. The first entry corresponds to YF_3_ and the second to HoF_3_. The mean of both values corresponds to the given order from top left to bottom right. Each (*hkl*) slab is rotated in a way to show the surface coordination best. For (111), two surfaces are given, as (111)-2 is preferred by YF_3_ and (111)-3 by HoF_3_.

**Table 2:** The YF_3_ (PBE) and HoF_3_ (PBE+U${}_{d}$/3 eV/4f-in-core) surfaces with respective terminations (term.), slab thickness in layers of formula units without terminal F-deficit ($L_{\mathrm{MF}3}$), nominal surface net charge ($q_{\mathrm{surf}}$) in e, surface energies of relaxed ($E_{\mathrm{surf}}$) and unrelaxed slabs ($E_{\mathrm{surf}}^{\mathrm{unrel}.}$) in J m${}^{-2}$, as well as the relaxed surface metal coordination number (CN${}_{\mathrm{surf}}$). The lowest surface energies per (hkl) cut are highlighted in bold. For these, also the abundance obtained by the Wulff plot (%${}_{\mathrm{surf}}$) is given.

			$L_{\mathrm{MF}3}$		CN${}_{\mathrm{surf}}$		$E_{\mathrm{surf}}$ ($E_{\mathrm{surf}}^{\mathrm{unrel}.}$)		%${}_{\mathrm{surf}}$	
**(** hkl **)**	**term.**	$q_{\mathrm{surf}}$	**YF_3_**	**HoF_3_**	**YF_3_**	**HoF_3_**	**YF_3_**	**HoF_3_**	**YF_3_**	**HoF_3_**
(100)	1	0	20	24	5,9		1.61 (2.87)	0.93 (1.48)		
	2	0	22	26	6,9		**1.03** (2.02)	**0.58** (0.96)	7%	25%
	3	+1	20	24	5,8		1.24 (1.61)	0.62 (0.68)		
	4	+2	22	26	4,7		1.79 (2.14)	0.87 (0.90)		
(010)	1	0	10	12	8,8		**0.58** (0.84)	**0.47** (0.49)	26%	34%
	2	+2	10	12	6,6		1.80 (2.05)	1.52 (1.52)		
(001)	1	0	20	24	5,8,8,9		1.23 (2.45)	1.37 (2.25)		
	2	0	22	26	6,7,8,9		**0.58** (1.39)	**0.67** (1.16)	10%	6%
	3	+2	22	26	4,5,8,9		1.27 (1.70)	1.23 (1.29)		
(110)	1	0	20	24	6,8,8		**1.01** (1.80)	**0.99** (1.59)	5%	0%
	2	0	22	26	6,8,8		**1.00** (2.41)	**1.00** (2.18)		
	3	+2	22	26	4,6,9	4,6,8	1.42 (1.73)	2.09 (1.36)		
(101)	1	0	20	24	6,7,8,8		0.82 (1.48)	0.89 (1.33)		
	2	0	20	24	6,6,8,8		0.82 (3.34)	0.88 (3.17)		
	3	+1	20	24	6,7,8,8		**0.76** (1.16)	**0.69** (0.89)	20%	14%
	4	+1	22	26	5,6,7,9	5,6,8,8	1.07 (2.10)	1.03 (1.70)		
	5	+2	20	24	4,5,8,8	5,6,8,8	0.98 (1.39)	0.99 (0.99)		
(011)	1	0	10	12	7,7,9,9		0.78 (1.30)	0.81 (1.14)		
	2	0	10	12	6,6,8,8		**0.61** (1.32)	**0.68** (1.15)	22%	13%
	3	+2	10	12	4,4,8,8		1.25 (1.68)	1.35 (1.38)		
(111)	1	0	20	24	6,7,7,8	7,7,8,8	1.02 (3.46)	0.87 (3.29)		
	2	+1	20	24	5,6,8,8		**0.83** (1.30)	0.82 (1.04)	10%	
	3	+1	22	26	6,6,7,9		1.05 (1.70)	**0.75** (1.11)		7%
	4	+2	20	24	5,5,7,7		0.93 (1.22)	0.95 (1.13)		
