# Supplementary material for: First Principle Surface Analysis of YF3 and Isostructural HoF3
Source: Materials (Basel). 2022 Sep 1;15(17):6048. doi: 10.3390/ma15176048 (PMC9457200; doi:10.3390/ma15176048)
Supplement: Supplementary file 1 [file materials-15-06048-s001.zip › materials-1875190-supplementary.pdf]

Supplementary Materials: First Principle Surface Analysis of β-YF_3-Structured Rare Earth Element Trifluorides

Jennifer Anders*, Niklas Limberg, Beate Paulus

Contents

1 Properties of the Bulk . . . 1
1.1 HoF_3 Bulk Benchmark . . . 1
1.2 Bulk Band Structures . . . 3
2 Relaxation Effect on Coordination Polyhedrons . . . 3
3 Convergence against Slab Thickness . . . 5
4 Error Estimation . . . 7
4.1 Error Estimation in Slab Thickness Convergence . . . 7
4.2 Error Estimation in Wulff Plots . . . 7
5 Electronic Properties of Surfaces . . . 8
5.1 Surface Band Gaps . . . 8
5.2 Surface DOS . . . 9

1. Properties of the Bulk

1.1. HoF_3 Bulk Benchmark

As for HoF_3, no experimental, but a purely empirically predicted band gap of ca. 9 eV exists, we calculated HSE06/4f-in-core and HSE06/4f-in-valence as references (Figure S1). Considering the computational demands and SCF convergence issues, only HSE06/4f-in-core was relaxed in unit cell parameters. The HSE06/4f-in-valence band gap is calculated on the experimental crystal structure. The difference between the two HSE06 direct band gaps is 3.24 eV. All 4f-in-core values of PBE and PBE+U_d with U = 1–7 eV are found within that range of both HSE06 values. However, all PBE+U_f/4f-in-valence band gaps stay below. Note the non-linear behavior of PBE+U_f/4f-in-valence at 6 eV. Here, the nature of the valence band maximum (VBM) changes from Ho-4f to F-2p. At 10 eV, the band structure collapses to a pseudo-metallic one.

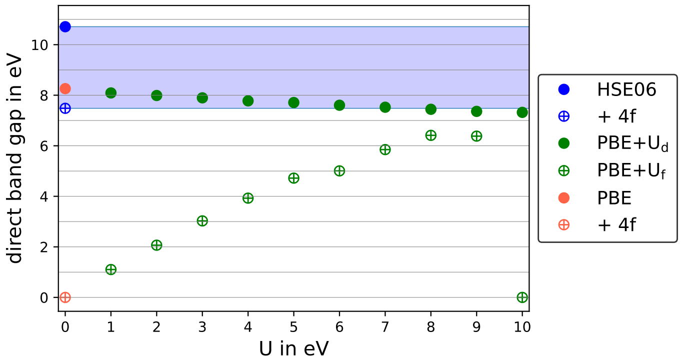

Figure S1. Calculated HoF_3 direct band gaps with HSE06 (blue), PBE+U (green) and pure PBE (red) applied on 4f-in-core (full markers) or 4f-in-valence (crosses); HSE06/4f-in-valence is not relaxed but done on-top of the crystal structure; the area between the two HSE06 values is highlighted in blue.

Pure PBE/4f-in-core performs already quite well on the band gap, as well as on the unit cell parameters (Figure S2). All PBE+U_f/4f-in-valence values perform worse with the exception of $U_f = 6$ eV. By increasing the potential in PBE+U_d, the unit cell parameters increase almost linearly up to $U_d = 8$ eV. At $U_d = 3$ eV, the relaxed unit cell volume

deviates by as little as 0.5% from the experimental value. HSE06/4f-in-core gives a much more shrunken unit cell, which is 15.6 Å^3 below the experimental value.

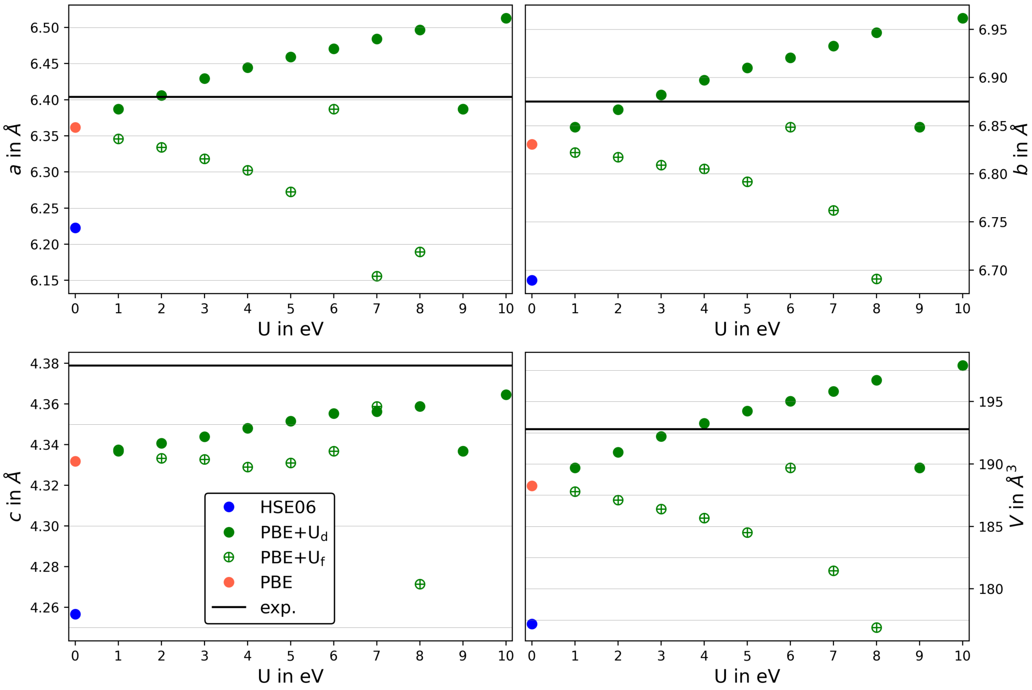

Figure S2. Calculated HoF_3 relaxed unit cell parameters with HSE06 (blue), PBE+U (green) and pure PBE (red) applied on 4f-in-core (full markers) or 4f-in-valence (crosses) compared to the experimental values (horizontal line).

In conclusion, the 4f-in-core approach reduces the computational demand and general SCF convergence issues inherent to the 4f-in-valence method, considerably. Moreover, it leaves the Bader charges practically unchanged and does not suffer from wrong spin arrangements (see main paper). In addition, it gives larger band gaps, which are closer to the predicted value and the calculated HSE06 ones. Finally, it yields the least structure derivation from experimental crystal structure (Figure S2 and main paper Table 1). Consequently, all HoF_3 slabs are obtained by PBE+U_d with 3 eV on 4f-in-core.

1.2. Bulk Band Structures

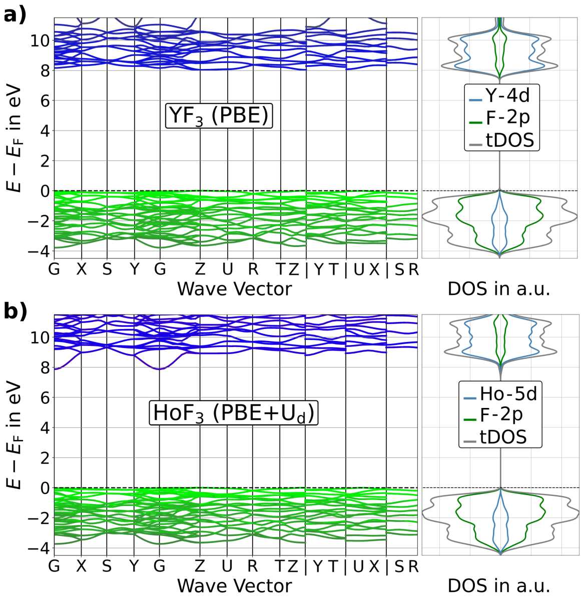

Figure S3. Bulk band structure, total DOS (tDOS: gray) and DOS projected onto the metal d band (blue) or fluorine 2p band (green): (**a**) YF_3 (PBE) and (**b**) HoF_3 (PBE+U_d/3 eV/4f-in-core).

Band structure calculations on the 3D-bulk show, that, typical for such ionic compounds, the bands are very localized or flat in k-space. This is especially true for the valence band (VB) of both compounds, which is mostly made from the 2p band of fluorine. The conduction band (CB) is mostly made from d band of holmium or yttrium. In YF_3, the CB is also very flat and featureless. In HoF_3, the CB has a slightly pronounced minimum (CBM) at the Γ-point.

2. Relaxation Effect on Coordination Polyhedrons

Table S1. Comparison of unrelaxed versus relaxed (or rearranged) slabs in metal coordination number at the surface (CN_{surf}), as well as in metal centers of the non-surface layers ($CN_{non\text{-}surf}$) as determined with a bond distance cut-off of 2.6 Å:

<table>
<tr><th colspan="2"></th><th colspan="4">unrelaxed</th><th colspan="4">relaxed</th></tr>
<tr><th></th><th></th><th colspan="2">$CN_{surf.}$</th><th colspan="2">$CN_{non\text{-}surf}$</th><th colspan="2">$CN_{surf.}$</th><th colspan="2">$CN_{non\text{-}surf}$</th></tr>
<tr><th>(hkl)</th><th>term.</th><th>YF_3</th><th>HoF_3</th><th>YF_3</th><th>HoF_3</th><th>YF_3</th><th>HoF_3</th><th>YF_3</th><th>HoF_3</th></tr>
<tr><td rowspan="4">(100)</td><td>1</td><td colspan="2">5,9</td><td colspan="2">9</td><td colspan="2">5,9</td><td colspan="2">9</td></tr>
<tr><td>2</td><td colspan="2">6,9</td><td colspan="2">9</td><td colspan="2">6,9 (2nd 8,8)</td><td colspan="2">9</td></tr>
<tr><td>3</td><td colspan="2">5,8</td><td colspan="2">9</td><td colspan="2">5,8</td><td colspan="2">9</td></tr>
<tr><td>4</td><td colspan="2">4,7</td><td colspan="2">9</td><td colspan="2">4,7 (2nd 8,9)</td><td colspan="2">9</td></tr>
<tr><td rowspan="2">(010)</td><td>1</td><td colspan="2">8,8</td><td colspan="2">9</td><td colspan="2">8,8</td><td colspan="2">9</td></tr>
<tr><td>2</td><td colspan="2">6,6</td><td colspan="2">9</td><td colspan="2">6,6</td><td colspan="2">9</td></tr>
<tr><td rowspan="3">(001)</td><td>1</td><td colspan="2">6,8,9,9</td><td colspan="2">9</td><td colspan="2">5,8,8,9</td><td colspan="2">8</td></tr>
<tr><td>2</td><td colspan="2">6,8,9,9</td><td colspan="2">9</td><td colspan="2">6,7,8,9</td><td colspan="2">8</td></tr>
<tr><td>3</td><td colspan="2">4,6,9,9</td><td colspan="2">9</td><td colspan="2">4,5,8,9</td><td colspan="2">8</td></tr>
<tr><td rowspan="3">(110)</td><td>1</td><td colspan="2">6,8,9</td><td colspan="2">9</td><td colspan="2">6,8,8</td><td colspan="2">9</td></tr>
<tr><td>2</td><td colspan="2">5,8,9</td><td colspan="2">9</td><td colspan="2">6,8,8</td><td colspan="2">9</td></tr>
<tr><td>3</td><td colspan="2">4,6,9</td><td colspan="2">9</td><td>4,6,9</td><td>4,6,8</td><td colspan="2">9</td></tr>
<tr><td rowspan="5">(101)</td><td>1</td><td colspan="2">6,8,8,9</td><td colspan="2">9</td><td colspan="2">6,7,8,8</td><td colspan="2">8</td></tr>
<tr><td>2</td><td colspan="2">4,6,9,9</td><td colspan="2">9</td><td colspan="2">6,6,8,8</td><td colspan="2">8</td></tr>
<tr><td>3</td><td colspan="2">6,8,8,8</td><td colspan="2">9</td><td colspan="2">6,7,8,8</td><td colspan="2">8</td></tr>
<tr><td>4</td><td colspan="2">5,6,8,9</td><td colspan="2">9</td><td>5,6,7,9</td><td>5,6,8,8</td><td colspan="2">8,9</td></tr>
<tr><td>5</td><td colspan="2">5,7,8,8</td><td colspan="2">9</td><td>4,5,8,8</td><td>5,6,8,8</td><td>8</td><td>9</td></tr>
<tr><td rowspan="3">(011)</td><td>1</td><td colspan="2">6,6,9,9</td><td colspan="2">9</td><td colspan="2">6,6,8,8</td><td colspan="2">8</td></tr>
<tr><td>2</td><td colspan="2">7,7,9,9</td><td colspan="2">9</td><td colspan="2">7,7,9,9</td><td colspan="2">8</td></tr>
<tr><td>3</td><td colspan="2">4,4,9,9</td><td colspan="2">9</td><td colspan="2">4,4,8,8</td><td colspan="2">8</td></tr>
<tr><td rowspan="4">(111)</td><td>1</td><td colspan="2">4,6,9,9</td><td colspan="2">9</td><td>6,7,7,8</td><td>7,7,8,8</td><td>8,(9)</td><td>8,9</td></tr>
<tr><td>2</td><td colspan="2">6,6,7,9</td><td colspan="2">9</td><td colspan="2">5,6,8,8</td><td colspan="2">8,(9)</td></tr>
<tr><td>3</td><td colspan="2">6,6,7,9</td><td colspan="2">9</td><td colspan="2">6,6,7,9</td><td colspan="2">8,9</td></tr>
<tr><td>4</td><td colspan="2">5,6,8,8</td><td colspan="2">9</td><td colspan="2">5,5,7,7</td><td colspan="2">8,9</td></tr>
</table>

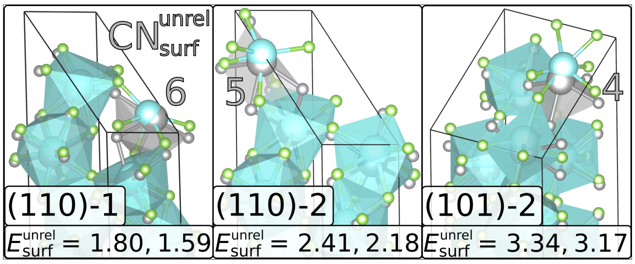

Figure S4. Effect of surface rearrangement on the stoichiometric surface terminations of (110)-1 (left), (110)-2 (middle) and (101)-2 (right). Atomic positions are shown before (gray) and after relaxation (M: blue, F: green). For the latter, all polyhedra are shown but the one from the initially lowest surface coordination number (CN^{unrel}_{surf}). Given are the surface energies in $J\,m^{-2}$ of the unrelaxed surfaces (E^{unrel}_{surf}) for YF_3 (first) and HoF_3 (second).

Table S1 shows the change in surface coordination numbers ($CN_{surf.}$). For three exemplary surfaces, these are also visualized in Figure S4. The 6-fold coordination polyhedron of (110)-1 reminds vaguely of a distorted pentagonal pyramid with the metal center cut by the pseudo-equatorial plane. Four out of five pseudo-equatorial fluorine have an angle of only 65–75° towards the axial fluorine. The 5-fold polyhedron of (110)-2 is obtained by removing one pseudo-equatorial fluorine from the 6-fold coordination p olyhedron. When allowed to rearrange in atomic positions, both terminations converged into an equivalent surface arrangement. Both show the same surface coordination and an identical surface energy within slab thickness convergence (Tables S2 and S3). In (101), the unrelaxed stoichiometric terminations (101)-1 and -2 mainly differ in a 4-fold versus 6-fold coordination (Figure S4 and Table S1). The latter is constructed as in (110)-1. Within the 4-fold polyhedron, all four fluorine point towards the second slab layer in a distorted 4-fold umbrella shape. After relaxation, (101)-1 and (101)-2, both, have six as their lowest coordination number and are also equivalent in surface energy. In contrast to (110), the relaxed polyhedron keeps a clear exposure of the metal ion similar to substoichiometric (101)-3 shown in main paper Figure 2.

Apart from the coordination at the surface layer, Table S1 also gives the coordination numbers of the non-surface metal centers ($CN_{non\text{-}surf}$). However, no correlation to E_{surf} could be found. During relaxation, the slabs expand in vacuum-direction. For some slabs, this leads to a reduction of some fully coordinated metal centers from 9 to 8 inside the non-surface layers. Within the non-surface layers of (111)-3 and -4, the 8-fold and 9-fold polyhedrons are both present in roughly the same ratio. Whereas in (111)-2, the 8-fold coordination strongly dominates within the non-surface layers. This is denoted by the parenthesis in Table S1. If only the coordination within the second layer is different from the other non-surface coordinations, as e.g. in (100)-2 and -4, this is denoted by (2nd). However, none of these changes in non-surface layers from 9-fold to 8-fold coordination does effect the Bader charges, discussed in the main paper (Figure 5).

Considering the surface layers, the very exposed $CN_{surf} = 4$ is only found for 4 (HoF_3) or 5 (YF_3) relaxed substoichiometric slabs missing two fluorine per surface. Initially, prior to relaxation, also stoichiometric (101)-2 and (111)-1 show a 4-fold coordination. Accordingly, their unrelaxed surface energies are among the highest ones. During relaxation, their surface energies reduce considerably while the surface coordination increases to $CN_{surf} = 6$.

3. Convergence against Slab Thickness

The bulk- and slab-derived surface energies of all calculated slab thicknesses are given in Table S2 for YF_3 and Table S3 for HoF_3. The respective stoichiometry is given in respect to the unit cell (UC) of M_4F_{12}.

Table S2. YF_3 (PBE) bulk-derived (E^{bd}_{surf}) and slab-derived (E^{sd}_{surf}) surface energies without (SP) and with atomic position relaxation (OPT); all energies in J m^{-2}; the $E^{bd}_{surf,opt}$ values are used within the main paper:

		SP		OPT	
(*hkl*)	stoichiometry	$E^{bd}_{surf,SP}$	$E^{sd}_{surf,SP}$	$E^{bd}_{surf,opt}$	$E^{sd}_{surf,opt}$
(100)-1	3 UC	2.72	—	1.56	—
	4 UC	2.79	2.50	1.59	1.47
	5 UC	2.87	2.51	1.61	1.48
(100)-2	4.5 UC	1.95	—	1.01	—
	5.5 UC	2.02	1.62	1.03	0.88
(100)-3	4 UC−2F	1.53	—	1.21	—
	5 UC−2F	1.61	1.25	1.24	1.11
(100)-4	4.5 UC−4F	2.07	—	1.76	—
	5.5 UC−4F	2.14	1.74	1.79	1.63
(010)-1	3 UC	0.68	—	0.51	—
	4 UC	0.76	0.45	0.54	0.40
	5 UC	0.84	0.45	0.58	0.40
(010)-2	4 UC−4F	1.97	—	1.77	—
	5 UC−4F	2.05	1.66	1.80	1.63
(001)-1	3 UC	2.35	—	1.25	—
	4 UC	2.40	2.20	1.24	1.28
	5 UC	2.45	2.20	1.23	1.26
(001)-2	4.5 UC	1.34	—	0.59	—
	5.5 UC	1.39	1.12	0.58	0.62
(001)-3	4.5 UC−4F	1.65	—	1.28	—
	5.5 UC−4F	1.70	1.42	1.27	1.31
(110)-1	3 UC	1.69	—	0.90	—
	4 UC	1.74	1.53	0.98	0.66
	5 UC	1.80	1.53	1.01	0.90
(110)-2	4.5 UC	2.35	—	0.98	—
	5.5 UC	2.41	2.11	1.00	0.88
(110)-3	4.5 UC−4F	1.68	—	1.40	—
	5.5 UC−4F	1.73	1.44	1.42	1.30
(101)-1	3 UC	1.40	—	0.78	—
	4 UC	1.44	1.27	0.81	0.72
	5 UC	1.48	1.27	0.82	0.76
(101)-2	3 UC	3.34	—	0.78	—
	4 UC	3.30	3.13	0.80	0.71
	5 UC	3.34	3.13	0.82	0.75
(101)-3	4 UC−2F	1.11	—	0.75	—
	5 UC−2F	1.16	0.95	0.76	0.68
(101)-4	4.5 UC−2F	2.06	—	1.07	—
	5.5 UC−2F	2.10	1.87	1.07	1.05
(101)-5	4 UC−4F	1.35	—	0.96	—
	5 UC−4F	1.39	1.18	0.98	0.89
(011)-1	3 UC	1.21	—	0.76	—
	4 UC	1.26	1.09	0.77	0.72
	5 UC	1.30	1.09	0.78	0.73
(011)-2	3 UC	1.23	—	0.59	—
	4 UC	1.27	1.10	0.60	0.56
	5 UC	1.32	1.10	0.61	0.57
(011)-3	4 UC−4F	1.64	—	1.24	—
	5 UC−4F	1.68	1.18	1.25	0.93
(111)-1	3 UC	3.37	—	0.59	—
	4 UC	3.42	3.24	1.00	-0.62
	5 UC	3.46	3.27	1.02	0.89
	6 UC	3.49	3.27	1.03	0.96
(111)-2	4 UC−2F	1.26	—	0.82	—
	5 UC−2F	1.30	1.11	0.83	0.77
(111)-3	4.5 UC−2F	1.66	—	1.03	—
	5.5 UC−2F	1.70	1.49	1.05	0.97
(111)-4	4 UC−4F	1.37	—	0.92	—
	5 UC−4F	1.40	1.22	0.93	0.85

Table S3. HoF_3 (PBE+U_d/3 eV/4f-in-core) bulk-derived (E^{bd}_{surf}) and slab-derived (E^{sd}_{surf}) surface energies without (SP) and with atomic position relaxation (OPT); all energies in J m^{-2}; all magnetic moments in μ_B; the $E^{sd}_{surf,opt}$ values are used within the main paper:

		SP			OPT		
(*hkl*)	stoichiometry	$E^{bd}_{surf,SP}$	$E^{sd}_{surf,SP}$	μ_{SP}	$E^{bd}_{surf,opt}$	$E^{sd}_{surf,opt}$	μ_{opt}
(100)-1	4 UC	1.47	—	0.00	0.77	—	0.00
	5 UC	1.46	1.48	0.00	0.73	0.93	0.00
	6 UC	1.46	1.48	0.00	0.69	0.93	0.00
	7 UC	1.46	1.48	0.00	0.66	0.93	0.00
(100)-2	4.5 UC	0.95	—	0.00	0.40	—	0.00
	5.5 UC	0.94	0.96	0.00	0.36	0.58	0.00
	6.5 UC	0.94	0.96	0.00	0.33	0.58	0.00
(100)-3	4 UC−2F	0.67	—	0.00	0.46	—	0.00
	5 UC−2F	0.67	0.68	0.00	0.43	0.62	0.00
	6 UC−2F	0.66	0.68	0.00	0.39	0.62	0.00
(100)-4	4.5 UC−4F	0.88	—	0.01	0.70	—	0.00
	5.5 UC−4F	0.88	0.90	0.00	0.66	0.88	0.00
	6.5 UC−4F	0.88	0.90	0.00	0.62	0.87	0.00
(010)-1	4 UC	0.47	—	0.00	0.22	—	0.00
	6 UC	0.45	0.49	0.00	0.10	0.47	0.00
	7 UC	0.45	0.48	0.00	0.05	0.39	0.00
(010)-2	4 UC−4F	1.51	—	4.03	1.28	—	3.53
	5 UC−4F	1.49	1.60	3.57	1.22	1.53	3.53
	6 UC−4F	1.48	1.52	3.57	1.16	1.52	3.53
(001)-1	4 UC	2.22	—	0.00	1.14	—	0.00
	5 UC	2.22	2.24	0.00	1.09	1.33	0.00
	6 UC	2.22	2.25	0.00	1.04	1.37	0.00
	7 UC	2.21	2.24	0.00	0.99	1.33	0.00
(001)-2	4.5 UC	1.15	—	0.00	0.43	—	0.00
	5.5 UC	1.14	1.16	0.00	0.38	0.67	0.00
	6.5 UC	1.14	1.16	0.00	0.33	0.67	0.00
(001)-3	4.5 UC−4F	1.24	—	0.00	0.95	—	0.00
	5.5 UC−4F	1.27	1.10	-0.01	0.93	1.08	0.00
	6.5 UC−4F	1.26	1.29	0.00	0.87	1.23	0.00
(110)-1	4 UC	1.57	—	0.00	0.81	—	0.00
	5 UC	1.56	1.58	0.00	0.76	1.00	0.00
	6 UC	1.56	1.59	0.00	0.71	0.99	0.00
	7 UC	1.55	1.59	0.00	0.66	1.01	0.00
(110)-2	4.5 UC	2.16	—	0.00	0.79	—	0.00
	5.5 UC	2.16	2.18	0.00	0.74	1.00	0.00
	6.5 UC	2.15	2.18	0.00	0.69	1.00	0.00
(110)-3	4.5 UC−4F	1.34	—	0.00	1.06	—	0.19
	5.5 UC−4F	1.34	1.36	0.47	1.01	1.27	0.27
	6.5 UC−4F	1.33	1.36	0.47	0.82	2.09	0.00
(101)-1	4 UC	1.32	—	0.00	0.75	—	0.00
	5 UC	1.14	1.33	0.00	0.72	0.87	0.00
	6 UC	1.13	1.33	0.00	0.69	0.89	0.00
	7 UC	1.13	1.33	0.00	0.65	0.90	0.00
(101)-2	4 UC	3.16	—	0.00	0.74	—	0.00
	5 UC	3.16	3.17	0.00	0.71	0.86	0.00
	6 UC	3.16	3.17	0.00	0.67	0.88	0.00
	7 UC	3.15	3.18	0.00	0.64	0.89	0.00
(101)-3	4 UC−2F	0.87	—	2.00	0.55	—	2.00
	5 UC−2F	0.87	0.88	2.00	0.52	0.68	2.00
	6 UC−2F	0.86	0.89	2.00	0.48	0.69	2.00
(101)-4	4.5 UC−2F	1.85	—	1.96	0.88	—	2.00
	5.5 UC−2F	1.84	1.87	2.00	0.84	1.05	2.00
	6.5 UC−2F	1.87	1.70	0.00	0.81	1.03	2.00
(101)-5	4 UC−4F	1.08	—	0.00	0.76	—	0.00
	5 UC−4F	1.08	1.09	0.00	0.74	0.83	0.00
	6 UC−4F	1.09	0.99	0.00	0.69	0.99	0.00
(011)-1	4 UC	1.12	—	0.00	0.65	—	0.00
	5 UC	1.12	1.14	0.00	0.62	0.81	0.00
	6 UC	1.12	1.14	0.00	0.58	0.81	0.00
	7 UC	1.12	1.14	0.00	0.54	0.79	0.00
(011)-2	4 UC	1.14	—	0.00	0.52	—	0.00
	5 UC	1.14	1.15	0.00	0.48	0.67	0.00
	6 UC	1.13	1.15	0.00	0.44	0.68	0.00
	7 UC	1.13	1.15	0.00	0.40	0.67	0.00
(011)-3	4 UC−4F	1.37	—	0.00	1.04	—	0.00
	5 UC−4F	1.36	1.38	0.00	1.01	1.19	0.00
	6 UC−4F	1.36	1.38	0.00	0.94	1.35	0.00
(111)-1	4 UC	3.27	—	0.00	0.72	—	0.00
	5 UC	3.25	3.36	1.95	0.72	0.71	0.00
	6 UC	3.24	3.29	0.00	0.70	0.87	0.00
	7 UC	3.25	3.23	1.84	0.67	0.88	0.00
(111)-2	4 UC−2F	1.03	—	2.00	0.70	—	2.00
	5 UC−2F	1.02	1.04	2.00	0.67	0.82	2.00
	6 UC−2F	1.02	1.04	2.00	0.64	0.82	2.00
(111)-3	4.5 UC−2F	1.46	—	2.00	0.86	—	2.00
	5.5 UC−2F	1.46	1.11	2.00	0.83	0.75	2.00
	6.5 UC−2F	1.46	1.11	2.00	0.80	0.75	2.00
(111)-4	4 UC−4F	1.15	—	0.04	0.79	—	0.00
	5 UC−4F	1.15	1.12	1.31	0.78	0.80	0.00
	6 UC−4F	1.16	1.13	2.92	0.75	0.95	0.00
	7 UC−4F	1.11	1.44	0.00	0.72	0.92	0.00

4. Error Estimation

The error in final total energy is maximum 10^{-6} eV. Compared to the the one in slab thickness convergence, this error is negligible. Of course, there might be considerable errors inherent to the applied electronic structure methods. However, these cannot be quantified without reference value. Therefore, we focus on the slab thickness convergence error.

4.1. Error Estimation in Slab Thickness Convergence

For YF_3, all surface energies slab-thickness-converged within 0.03 J m^{-2} at thicknesses of about 5–5.5 UC. For HoF_3, 14 terminations including all of the most stable ones per Miller indices converged to 0.01 J m^{-2} or less within slab thickness of about 6–6.5 UC. Some of the higher energy terminations converged only to 0.02–0.04 J m^{-2} at that thickness, whereas four high energy terminations did not converge even to 0.1 J m^{-2}. The difference in surface energy between the two largest adjacent slab thickness are visualized as error bars in Figure S5.

In HoF_3, the surface energy of (110)-3 with a fluorine-deficit of two per surface is much higher with 2.09 J m^{-2} than any other. It also contains the highest uncertainty due to slab thickness convergence as shown in Table S3 and Figure S5. The large difference to the next smaller slab thickness seems to correlate with the change in magnetic structure from $\mu_{opt} = 0.27\,\mu_B$ to none.

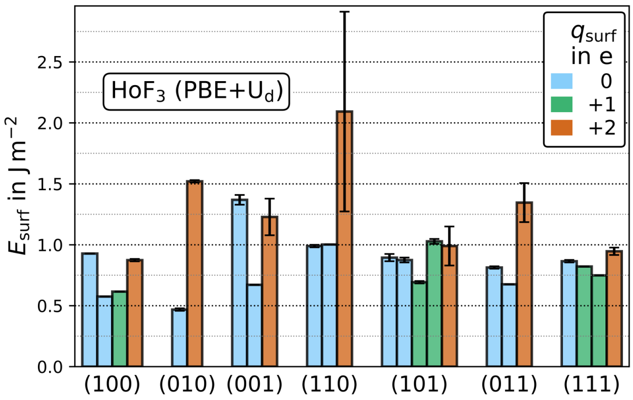

Figure S5. Relaxed slab-derived surface energies of HoF_3 (PBE+U_d/3 eV/4f-in-core). The uncertainty due to slab thickness convergence is given by error bars on each termination.

4.2. Error Estimation in Wulff Plots

The Wulff plot is constructed by the lowest energy termination of each Miller indices. These have a slab thickness convergence error of maximum 0.03 J m^{-2} or 0.01 J m^{-2} for YF_3 or HoF_3, respectively. The error margins for the Wulff plots given in Table S4 come from a very conservative view and give the maximum of possible error accumulation. For the very tiny surface percentages this gives huge relative errors of 46–100%. For the two most important surfaces, with 25% or 34% surface abundance, the relative errors are 8% or 12%. Note that, due to the geometrical interdependence of the surfaces, the absolute errors are not simply symmetrical around each initial value, but might be generally over- or underestimating.

Table S4. Effect of maximal error accumulation due to the convergence in slab thickness of maximal $\pm 0.03\,\mathrm{J\,m^{-2}}$ for YF_3 and $\pm 0.01\,\mathrm{J\,m^{-2}}$ for HoF_3 onto Wulff construction; i denotes the initial value of average surface energy ($\varnothing E_{surf}$) or surface abundance ($\%_{surf}$) given by the Wulff plots in the main paper Figure 4:

	YF_3		HoF_3	
	i	± 0.03	i	± 0.01
$\varnothing E_{surf}$ in $\mathrm{J\,m^{-2}}$	0.70	0.66–0.73	0.59	0.57–0.60
$\%_{surf}(100)$	7	4–10	25	25–27
$\%_{surf}(010)$	26	21–30	34	32–36
$\%_{surf}(001)$	10	5–17	6	5–8
$\%_{surf}(110)$	5	2–10	0	0
$\%_{surf}(101)$	20	11–29	14	11–18
$\%_{surf}(011)$	22	12–33	13	10–16
$\%_{surf}(111)$	10	2–23	7	4–11

5. Electronic Properties of Surfaces

5.1. Surface Band Gaps

The direct and indirect band gaps of all slabs are given in Figure S6. It should be noted, that these values are directly obtained from the k-point grid of $9 \times 9 \times 1$ for YF_3 or $7 \times 7 \times 1$ for HoF_3. No band structures have been calculated for the 2D-slab models. In agreement with the rather flat band structures of bulk YF_3 and HoF_3 shown in Figure S3, most slabs also show a direct Γ–Γ band gap. The Γ-point is included within our k-grids. However, some show indirect band gaps including a k-point, which is not explicitly included within the k-grid. For these, the actual band gaps might slightly differ from the ones given.

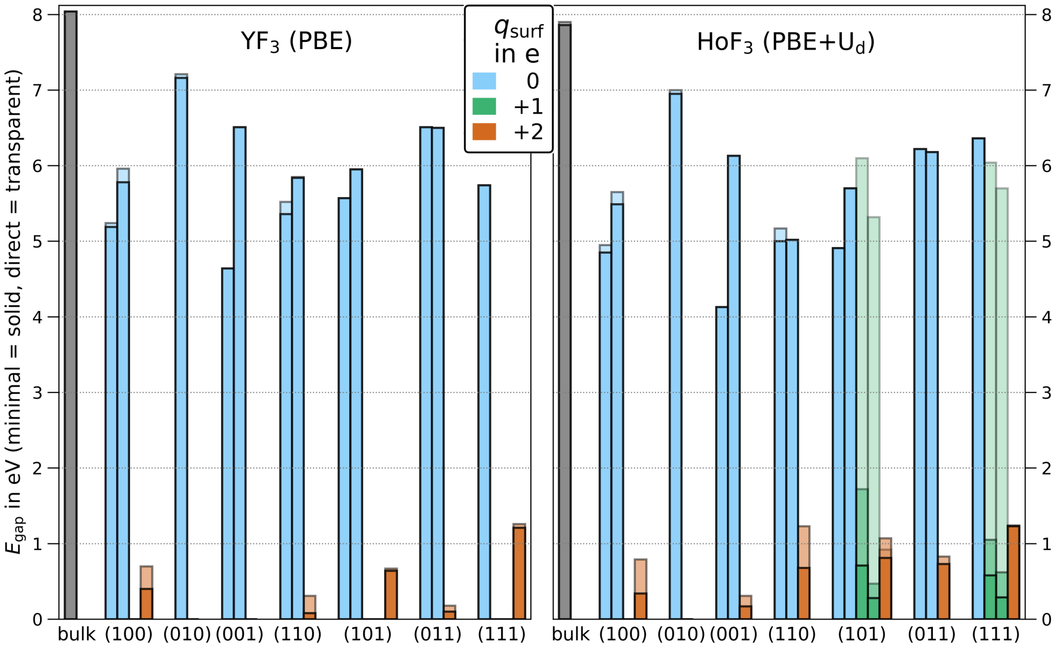

Figure S6. YF_3 (left, PBE) and HoF_3 (right, PBE+U_d/3 eV/4f-in-core) band gaps of surfaces compared with the respective bulk value (gray). Minimal band gaps, direct or indirect are given by solid bars. In the case, the minimal band gap was found to be indirect, also the direct band gap is given by a transparent bar. For HoF_3 (101) and (111) with +1 nominal charges, the band gaps are not spin-symmetric and both direct transitions are given.

5.2. *Surface DOS*

Slab convergence was tested against the direct band gaps, total DOS and projected DOS onto central-slab atoms. We found that the valence band and near conduction band are already converged at our smallest slab sizes. A comparison of the total DOS between the most stable termination of each (*hkl*) is shown in Figure S7.

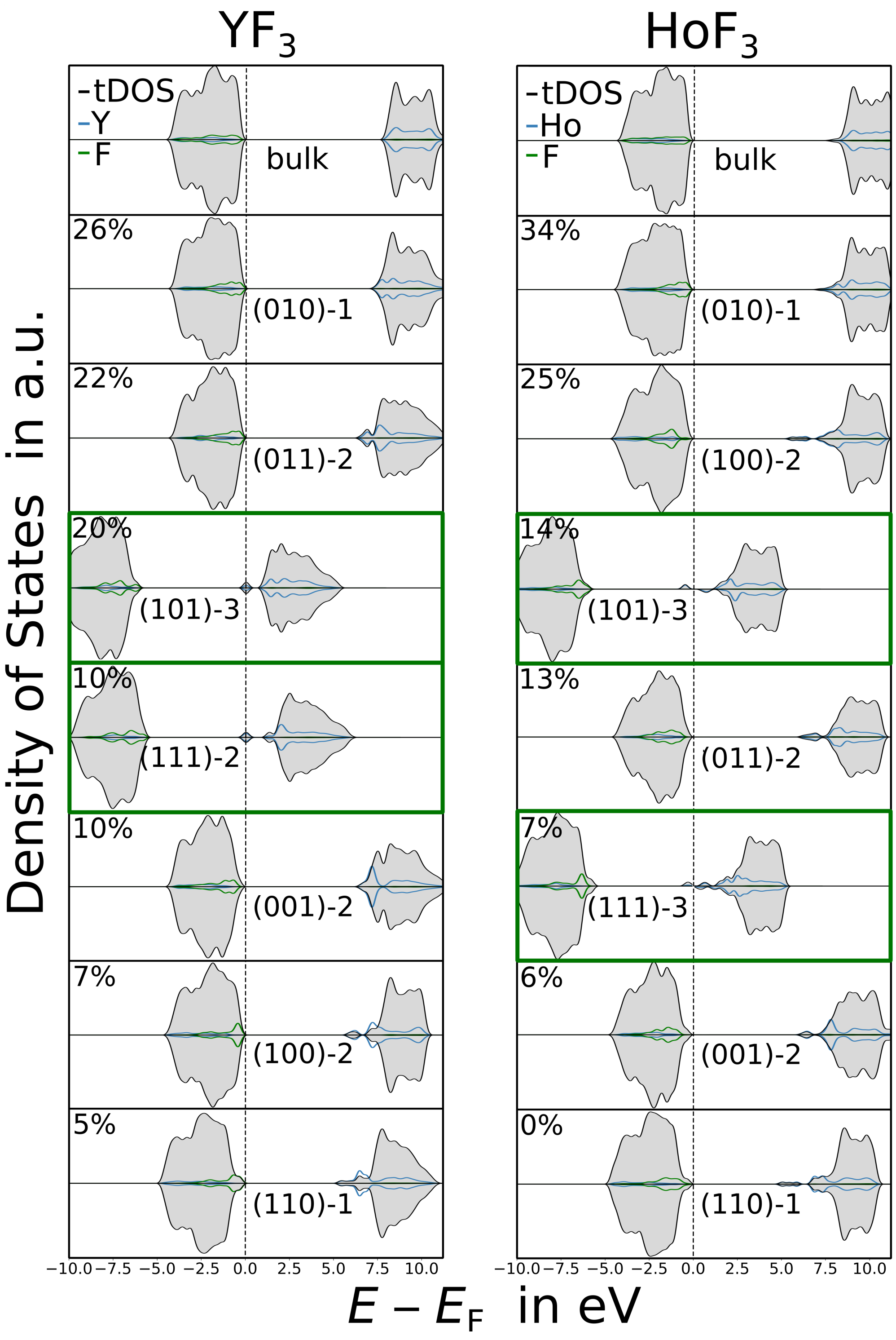

Figure S7. DOS comparison between the most stable surfaces ordered by their abundance (in %): YF_3 (left, PBE), HoF_3 (right, PBE+U_d/3 eV/4f-in-core), total DOS (tDOS: gray, downscaled to the bulk tDOS) and projected DOS of a single surface atom (Y, Ho: blue; F: green). Substoichiometric slabs with a fluorine-deficit of 1 per surface are framed in green. The top row gives the bulk tDOS with projected DOS of a single bulk atom as reference.
